# DropDAE: Denosing Autoencoder with Contrastive Learning for Addressing Dropout Events in scRNA-seq Data

**DOI:** 10.3390/bioengineering12080829

**Published:** 2025-07-31

**Authors:** Wanlin Juan, Kwang Woo Ahn, Yi-Guang Chen, Chien-Wei Lin

**Affiliations:** 1 Division of Biostatistics, Data Science Institute, Medical College of Wisconsin (MCW), Milwaukee, WI 53226, USA; wjuan@mcw.edu (W.J.); kwooahn@mcw.edu (K.W.A.); 2 Department of Pediatrics, Medical College of Wisconsin (MCW), Milwaukee, WI 53226, USA; yichen@mcw.edu

**Keywords:** dropout, imputation, autoencoder, denoising autoencoder, scRNA-seq, deep learning

## Abstract

Single-cell RNA sequencing (scRNA-seq) has revolutionized molecular biology and genomics by enabling the profiling of individual cell types, providing insights into cellular heterogeneity. Deep learning methods have become popular in single cell analysis for tasks such as dimension reduction, cell clustering, and data imputation. In this work, we introduce DropDAE, a denoising autoencoder (DAE) model enhanced with contrastive learning, to specifically address the dropout events in scRNA-seq data, where certain genes show very low or even zero expression levels due to technical limitations. DropDAE uses the architecture of a denoising autoencoder to recover the underlying data patterns while leveraging contrastive learning to enhance group separation. Our extensive evaluations across multiple simulation settings based on synthetic data and a real-world dataset demonstrate that DropDAE not only reconstructs data effectively but also further improves clustering performance, outperforming existing methods in terms of accuracy and robustness.

## 1. Introduction

Single-cell RNA sequencing (scRNA-seq) has revolutionized molecular biology and genomics by enabling the exploration of cellular heterogeneity. Unlike traditional bulk RNA-seq, which provides averaged gene expression levels across a cell population [1], scRNA-seq captures the unique transcriptomes of single cells, offering valuable insights into cellular heterogeneity.

Despite its advantages, scRNA-seq data are inherently more noisy than bulk RNA-seq data due to dropout events, where certain genes exhibit very low or zero expression levels because of technical limitations [2]. These dropouts contribute to data sparsity and zero inflation, complicating downstream analyses. Standard statistical imputation approaches typically assume that missing values are missing at random or missing completely at random. However, this assumption cannot be applied in scRNA-seq data since genes with lower expression are more likely to suffer from dropout events, requiring more flexible denoising or imputation methods without such assumptions.

In the literature, dropout events in scRNA-seq data have been interpreted in multiple ways. Many methods treat dropouts as missing values and apply imputation approaches to recover unobserved expression levels [3,4]. Others view dropouts as noise and aim to denoise the entire dataset using global models [5]. More recently, an alternative perspective has emerged in which dropouts are considered to carry biologically meaningful signals [6]. These diverse interpretations reflect the complexity of dropout mechanisms and motivate the development of flexible methods that can accommodate multiple assumptions.

Dropout events significantly impact downstream analyses and the interpretation of scRNA-seq data. Various methods have been developed to address dropouts, broadly classified into two categories. Methods in the first category borrow information from similar cells or genes, typically defined by distance metrics or clustering co-membership. For example, CCI [3] utilizes consensus clustering to identify similar cells and leverages this information to impute gene expression levels. RESCUE [4] employs bootstrap sampling from neighboring cells to estimate gene expression distributions and imputes using the distribution means. Methods in the second category use global model-based approaches to denoise the entire dataset, which often use parametric models. For instance, DCA [5] employs a deep autoencoder framework with a zero-inflated negative binomial loss function to recover the gene expression data patterns. However, existing methods have notable disadvantages. First-category methods need to define the neighboring cells or genes and often use sampling methods, resulting in heavy computational burdens, especially for datasets with large numbers of cells. Second-category methods may rely on parametric assumptions about data distribution, potentially compromising effectiveness if these assumptions are not satisfied [5]. Furthermore, current evaluations frequently depend on specific simulation settings, limiting comparative effectiveness across different methods and raising concerns about their generalization to various scenarios [4,7,8].

Deep learning methods have gained popularity in single-cell analysis for tasks including dimensionality reduction [9], cell clustering [10,11], data imputation [5], trajectory analysis, and batch effect correction. These models offer several advantages over traditional approaches, including their higher computing efficiency; that they do not necessarily require parametric assumptions; and that they are highly flexible in handling complex and noisy data. Among the various deep learning architectures, Denoising Autoencoders (DAEs) [12] have shown particular promise in handling noise and contamination in the data. By learning to reconstruct original, uncorrupted input data from corrupted versions, DAEs capture robust representations that reflect the underlying data structure. This denoising capability makes them suitable for imputation tasks, where the goal is to recover missing or distorted information. However, standard DAEs may not fully capture the compex structure of scRNA-seq data or effectively separate diverse cell populations. In particular, their application to effectively address dropout events in scRNA-seq data remains underexplored.

To address the limitations of existing approaches, we propose DropDAE, a Denoising Autoencoder (DAE) enhanced with contrastive learning [13], specifically designed to impute dropout events in scRNA-seq data. DropDAE leverages the DAE architecture to learn how to effectively remove noises from corrupted input data and recover the underlying data structure. To further improve representation learning, contrastive learning is integrated to promote better group separation and enhance clustering performance. Section 2 provides an overview and mathematical details of DropDAE, settings of simulation studies, implementation of competing methods, and quantitative assessment methods. Section 3 demonstrates the performance of DropDAE against competing methods using both simulated and real-world datasets. We summarize our findings in Section 5 and provide a discussion in Section 4.

## 2. Methods

### 2.1. Overview of DropDAE Method

**Figure 1:**
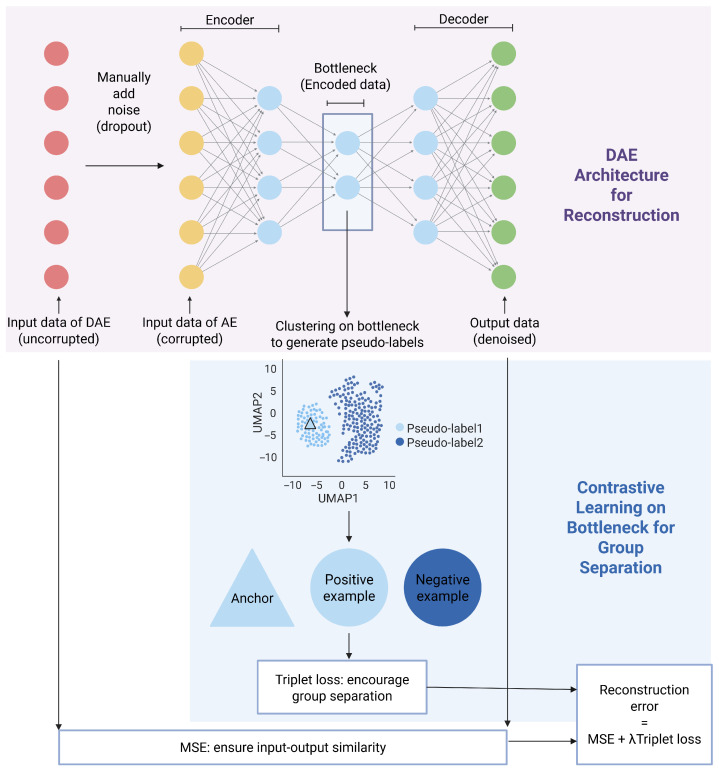
Pipeline of DropDAE. Created in BioRender. Juan, W. (2025) https://BioRender.com/k0128u5, accessed 5 June 2025. DropDAE denoises the scRNA-seq data by learning representations that maps noisy gene expression profiles back to their true underlying structure. The denoising autoencoder consists of an input layer, followed by a symmetric autoencoder structure with matching encoder and decoder sizes. The red nodes represent the original uncorrupted data, while the yellow nodes represent manually corrupted inputs by additional dropouts. The blue nodes in the bottleneck depict the latent variables, which are the lower-dimensional representations of the input data used for contrastive learning. The green nodes indicate the reconstructed outputs, where the model recovers the original data patterns. DAE is trained by minimizing the reconstruction error using MSE between the uncorrupted input and the denoised output and triplet loss calculated using pseudo-labels defined by bottleneck, enhancing both data reconstruction and group separation.

An overview of the DropDAE method is illustrated in Figure 1, where the core of the algorithm is based on a DAE framework, composed of an input layer, an encoder, and a decoder. Unlike a standard autoencoder (AE), DAE deliberately corrupts the input data (depicted as red nodes) by introducing extra noise in the input layer, ensuring that the autoencoder captures effective lower-dimensional representation to avoid identity transformation from the original data. Similarly, DropDAE artificially generates a corrupted version of input $\tilde{x}$ by introducing additional dropout events to the original input *x* using the R function *splatSimDropout* in R package *Splatter* [14]. This function can be used to control dropout distribution parameters, enabling realistic dropout scenarios by specifying the parameter *dropout.mid*, which controls the probability that a particular gene count in a cell is set to zero. Only mild dropout corruption is introduced to encourage the network to learn meaningful dropout patterns while avoiding trivial identity mapping.

DropDAE then feeds the corrupted input data (depicted as yellow nodes in Figure 1) into the AE, which holds the symmetric structure, consisting of an encoder $f_{\theta }\left(\cdot \right)$ and a decoder $g_{\phi }\left(\cdot \right)$. The encoder compresses the corrupted input data into a low-dimensional representation (bottleneck, depicted as blue nodes) by learning only the essential features. Mathematically, it maps the corrupted input data $\tilde{x}$ to a latent variable *z* through function $f_{\theta }\left(\cdot \right)$ parameterized by θ: $z=f_{\theta }\left(\tilde{x}\right)$. This latent representation *z*, termed the bottleneck, captures the critical data structure, facilitating dimension reduction and serving as the basis for defining pseudo-labels in contrastive learning. The decoder reconstructs the original input data from this low-dimensional representation. It takes the latent variable *z* and maps it back to the original data space through function $g_{\phi }\left(\cdot \right)$ parameterized by ϕ: $\hat{x}=g_{\phi }\left(z\right)$. The dimensions of DropDAE layers can be specified, while the default configuration is 64 units for the encoder layer, 32 units for the bottleneck layer, and 64 units for the decoder layer. Weight decay (L2 regularization) is applied following the recommendations in [15], and all hidden layers use Rectified Linear Unit (ReLU) activation functions.

Typically, DAEs measure reconstruction accuracy through mean squared error (MSE), ensuring similarity between the reconstructed output $\hat{x}$ and the original uncorrupted data x as follows:

$$\mathrm{MSE}\left(x,\hat{x}\right)=\frac{1}{G}{\left(x_{i}-{\hat{x}}_{i}\right)}^{2},$$

where *G* denotes the number of genes.

However, in scRNA-seq data analysis, high reconstruction accuracy alone may not suffice, since clustering cells into different subtypes is a major downstream goal. To enhance cluster separation, DropDAE integrates contrastive learning, a representation learning technique that maps data into a feature space where similar clusters are nearby and dissimilar ones are further apart [13]. While contrastive learning is traditionally used in supervised contexts, it can be effectively applied in unsupervised settings by generating pseudo-labels through clustering [16,17].

DropDAE employs triplet loss, a popular contrastive learning loss function [18], in an unsupervised manner: in each training epoch, DropDAE performs clustering analysis on the bottleneck latent representations to generate and update pseudo-labels (see details in Section 2.2). Within each batch, cells act as anchors, and for each anchor cell, one positive example (from the same predicted cluster) and one negative example (from a different predicted cluster) are selected randomly. Triplet loss optimizes the distances between triplets of samples: one anchor, one positive sample, and one negative sample. It minimizes the distance between the anchor and the positive while maximizing the distance to the negative, encouraging cluster separation in latent space:

$$\mathrm{Triplet}\;\mathrm{loss}=max\left({0,∥f\left(A\right)-f\left(P\right)∥}^{2}-{∥f\left(A\right)-f\left(N\right)∥}^{2}+\alpha \right),$$

where f(·) denotes the embedding function learned by the autoencoder, and A, P, and N denote the anchor cell, positive sample, and negative sample, respectively. The margin parameter α enforces a minimum separation between the anchor–positive and anchor–negative pairs. The notation ||·|| denotes the euclidean distance in the latent space.

The overall loss function in DropDAE combines MSE and triplet loss to balance input–ouput similarity with improved cluster differentiation as follows:

Totalloss=MSE+λTripletloss,

where the hyperparameter λ controls the relative importance of the triplet loss, directly influencing the effectiveness of clustering.

In summary, the following steps for DropDAE are performed sequentially:

1. Corrupt the input scRNA-seq expression matrix by manually adding dropout noise.
2. Pass the corrupted input through the encoder of denoising autoencoder to obtain the latent representation (bottleneck) *z*.
3. Perform a clustering method (e.g., *K*-means or consensus clustering) on the latent representation *z* to generate pseudo-labels.
4. Sample anchor–positive–negative triplets from the latent space based on the pseudo-labels. Compute the triplet loss to encourage separation.
5. Pass the bottleneck *z* to the decoder of the denosing autoencoder to obtain the reconstructed output. Compute the mean squared error (MSE) between the reconstructed and original input.
6. Compute the total loss as the weighted sum of MSE and triplet loss. Update the model parameters via backpropagation.

DropDAE training employs mini-batch gradient descent, updating model parameters iteratively with specified batch sizes. The model is trained over multiple epochs with a fixed or adaptive learning rate. To further enhance model stability and generalizability, DropDAE incorporates early stopping, halting training if the total loss does not improve for a predefined number of epochs, named patience, preventing overfitting and reducing computational load. The repeat times of training per dataset can also be specified, then the best-performing model with the lowest loss will be selected as the final solution. The margin α and the weighting parameter λ are also critical hyperparameters, carefully tuned to balance effective reconstruction with meaningful clustering.

Two key hyperparameters in DropDAE are the margin α used in the triplet loss and the weighting parameter λ that balances the reconstruction (MSE) and contrastive (triplet) loss. These parameters critically influence the trade-off between data fidelity and latent space separability. In our study, we conducted a comprehensive tuning procedure to determine robust values for these hyperparameters. Specifically, we evaluated multiple combinations of α∈{1,5,10,20} and λ∈{1,5,10,20} across nine simulation scenarios defined by a factorial design of three dropout levels and three differential expression signal levels using two-group data. For each combination, we assessed clustering performance using ARI and compactness, selecting the hyperparameters that yielded the best overall performance across settings. As a result, we chose α=10 and λ=10 for all subsequent experiments. This configuration provided a strong balance between accurate gene expression reconstruction and meaningful cell group separation in the latent space, and it demonstrated robust performance across both simulated and real datasets.

Overall, this systematic approach in DropDAE ensures both accurate reconstruction of gene expression data and enhanced clustering performance, producing robust and biologically meaningful representations suitable for downstream analytical tasks.

### 2.2. Clustering on the Bottleneck Latent Representations: *K*-Means and Consensus Clustering

In the DropDAE framework, clustering plays a central role in contrastive learning by providing pseudo-labels used to form triplets for training. Specifically, we use both *K*-means and consensus clustering to determine cell groupings based on the latent representations *z* obtained from the bottleneck layer of the autoencoder.

*K*-means is a widely used unsupervised clustering algorithm that partitions a dataset of *n* observations into *K* disjoint clusters by minimizing the within-cluster sum of squared distances. Formally, given a dataset $\left\{x_{1},x_{2},\ldots ,x_{n}\right\}$, *K*-means solves the following optimization problem:

$$\underset{C_{1},\ldots ,C_{K}}{\arg\;\min}\sum_{k=1}^{K}\sum_{x_{i}\in C_{k}}{∥x_{i}-\mu_{k}∥}^{2},$$

where $C_{k}$ denotes the set of points in cluster *k*, and $\mu_{k}$ is the centroid of cluster *k*. In our implementation, prior to applying *K*-means, we first perform dimensionality reduction using Principal Component Analysis (PCA) followed by Uniform Manifold Approximation and Projection for Dimension Reduction (UMAP) [19] to project the high-dimensional data into a lower-dimensional space. The notation ||·|| denotes the euclidean distance in the latent space. This step avoids performing *K*-means directly on the high-dimensional expression matrix and improves both computational efficiency and clustering accuracy. *K*-means is implemented via the kmeans function in the R package *stats*.

To increase robustness and reduce sensitivity to random initialization or single clustering errors, we also employ consensus clustering. This approach aggregates clustering results from multiple subsampled datasets to form a stable consensus label for each cell. Mathematically, for each pair of cells (i,j), we define the consensus score $s_{ij}$ as the proportion of clustering runs in which cells *i* and *j* are assigned to the same cluster as follows:

$$s_{ij}=\frac{1}{T}\sum_{t=1}^{T}I\left\{c^{\left(t\right)}\left(i\right)=c^{\left(t\right)}\left(j\right)\right\},$$

where $c^{\left(t\right)}\left(i\right)$ is the cluster label of cell *i* in the *t*-th clustering run and *T* is the total number of runs. The notation I· denotes the indicator function. Given a threshold *c*, we define a pair as belonging to the same cluster in the consensus matrix if $s_{ij}>c$. This helps mitigate the effects of instability in individual clustering results and supports more reliable positive and negative pair selection in contrastive learning.

Importantly, this clustering is applied to the latent embedding space *z* learned by the bottleneck layer of the autoencoder, not to the raw or imputed gene expression matrix. Its primary purpose is to support contrastive learning during training by generating pseudo-labels, rather than to serve as a final evaluation tool.

In Section 3, we apply *K*-means primarily for speed and its ability to provide quick pseudo-label updates during training. Consensus clustering is applied post-training or in triplet selection steps to refine pair assignment and improve label consistency. These pseudo-labels are only used for constructing triplets and computing triplet loss, rather than benchmarking downstream performance.

After imputation, we use shared nearest neighbor (SNN) graph clustering [20] to evaluate final performance, aligning with standard evaluation practices used by competing methods. This final clustering is performed on the denoised expression matrix output by the decoder.

### 2.3. Simulation Settings

To comprehensively evaluate the performance and robustness of DropDAE against competing methods, we simulate scRNA-seq datasets using the *splatSimulateGroups* function from the R package *Splatter* [14]. This function allows for control over both differential expression (DE) signals and dropout parameters, making it highly suitable for simulating realistic scRNA-seq data scenarios. Specifically, we simulate two and six distinct cellular groups, adjusting the similarity between these groups by tuning the differential expression parameter de.facLoc. This parameter modulates the differential expression of selected genes, where lower values correspond to weaker differential expression signals, leading to higher similarity between the simulated groups.

To model varying dropout levels, we utilize the parameter dropout.mid, which influences the likelihood that individual gene counts in cells are artificially set to zero. The dropout process is modeled using a logistic relationship dependent on the gene’s mean expression within each cell. Lower values of dropout.mid result in fewer dropout events, reflecting weaker dropout conditions.

In our simulations, we systematically explore three distinct levels of DE signal strengths and three corresponding levels of dropout severity. For differential expression, we set de.facLoc to values of 0.3, 0.2, and 0.1, representing strong, moderate, and weak differential expression signals, respectively. Similarly, we adjust dropout.mid to 5, 4, and 2 to reflect strong (76%), moderate (62%), and weak (32%) dropout rates, respectively.

Each simulated dataset consists of 1000 cells per group with two or six groups, with measurements for 500 genes. To ensure statistical reliability and robustness, we replicate each simulation setting 200 times. The performance metrics for each downstream analysis are averaged across these repetitions, facilitating an objective assessment of each method’s effectiveness and stability. Additional parameters employed in the simulation process are summarized for clarity in Table 1.

All experiments were conducted on the High-Performance Computing (HPC) cluster at the Medical College of Wisconsin. For our analyses, we typically utilized compute nodes with 12 CPU cores and 90 GB of RAM. The DropDAE model was implemented in R using the torch package, which supports GPU acceleration, though all reported results were obtained using CPU execution only.

Across our simulated datasets with 500 genes and 2000 cells in 2 groups, the computational time for DropDAE costs approximately 9 min per dataset using 300 epoches with 1 initialization. Competing methods were executed using their recommended settings under similar resource constraints. All runs used fixed seeds to ensure reproducibility.

### 2.4. Implementation of Competing Methods

To benchmark the performance of DropDAE, we compare it against two representative imputation methods: RESCUE and DCA. These methods were selected as they reflect the two main categories of dropout handling approaches introduced in Section 1. Specifically, RESCUE represents methods that rely on borrowing information from similar cells (category one), while DCA exemplifies methods that apply global denoising strategies based on parametric models (category two).

RESCUE [4] leverages bootstrap-based sampling to estimate gene expression distributions by calculating the average expression across randomly sampled cells. We implement RESCUE using the bootstrapImputation function from the R package *rescue*. Input data for RESCUE must be preprocessed by normalization. After obtaining imputed values, we scale the data without performing any further normalization since the original normalization step has already been completed. All the parameters and options remain at their standard settings.

DCA [5] utilizes an autoencoder framework employing a zero-inflated negative binomial (ZINB) likelihood to denoise scRNA-seq data. We use the original Python package *dca* to implement DCA, explicitly setting the loss function to ZINB following guidelines outlined by the method’s authors. All other parameters for the DCA implementation are maintained at their default configurations.

### 2.5. Evaluation of Downstream Analyses

We present a comprehensive evaluation framework to assess the clustering performance of DropDAE. In scRNA-seq analysis, clustering accuracy and structure are crucial for interpreting the biological relevance of latent representations.

To visualize clustering patterns, we apply standard dimensionality reduction techniques including Principal Component Analysis (PCA), Uniform Manifold Approximation and Projection (UMAP) [19], and t-distributed Stochastic Neighbor Embedding (t-SNE) [21]. These are generated using the RunPCA, RunUMAP, and RunTSNE functions from the R package *Seurat*.

Quantitative assessment is conducted using the Adjusted Rand Index (ARI) [22], which measures the similarity between predicted clustering results and true labels. ARI adjusts for chance agreement and takes values from 0 to 1, with 1 indicating perfect clustering correspondence and values near 0 suggesting random labeling.

To further characterize the quality of clustering beyond ARI, we calculate compactness [3], which reflects the separation between identified clusters. Compactness is defined as the ratio of between-cluster variance to the total variance, providing insight into the tightness and distinctiveness of clusters. Higher compactness values indicate clearer separation among groups.

Let *K* be the number of clusters, $n_{k}$ the number of observations in cluster *k*, ${\bar{x}}_{k}$ the centroid of cluster *k*, $\bar{x}$ the overall centroid, and $x_{i}$ the feature vector for an observation belonging to cluster *k*. The notation ||·|| denotes the euclidean distance in the latent space. The between-group variance *B*, within-group variance *W*, and compactness are calculated as follows:

$$B=\sum_{k=1}^{K}n_{k}∥{\bar{x}}_{k}-\bar{x}{∥}_{2}^{2},\;W=\sum_{k=1}^{K}\sum_{c(i)=k}{∥x_{i}-{\bar{x}}_{k}∥}_{2}^{2},\;\mathrm{compactness}=\frac{B}{B+W}.$$

Together, ARI and compactness offer a complementary evaluation strategy that captures both the accuracy of clustering assignments and the quality of group separation.

While some imputation studies evaluate performance at the single-gene level [3], we intentionally focus on clustering-based evaluation in this study. DropDAE is a denoising method, designed to reconstruct the global structure of the gene expression matrix rather than merely imputing zero counts caused by dropout. As such, it modifies a wide range of expression values to enhance biological signal and suppress noise. Therefore, metrics like MSE or RMSE, which penalize all deviations from the original matrix regardless of biological relevance, may underestimate the utility of denoising models like DropDAE or competing method DCA. In contrast, clustering reflects the primary objective of many scRNA-seq analyses and is a more appropriate metric for assessing the effectiveness of denoising methods.

## 3. Results

### 3.1. DropDAE Restores and Improves the Original Clustering Pattern

We evaluate the performance of DropDAE using simulation studies designed to assess its denoising accuracy and enhancement in clustering. Synthetic single-cell data are generated using the R package *Splatter* [14], with varying levels of differential expression (DE) signals (weak, moderate, and strong) as ground truths. Dropout noises are added at different intensities (weak, moderate, and strong) to mimic realistic technical artifacts in scRNA-seq data. These dropout-affected data serve as inputs for DropDAE and competing imputation methods, including RESCUE and DCA, which belong to the first and the second category, respectively, in Section 1. Details of the simulation parameters and method implementations are described in Section 2.3 and Section 2.4, respectively.

To assess clustering performance, we follow a standardized evaluation pipeline. After applying each method to the input data, we perform dimensionality reduction, followed by clustering with the shared nearest neighbor (SNN) graph-based algorithm [23]. This approach, implemented in the *Seurat* package, constructs a graph where cells are connected based on shared neighborhoods in the reduced space, then it applies community detection like the Louvain algorithm to identify clusters. The SNN-based method is robust to noise, scales well to large datasets, and is agnostic to the imputation or denoising strategy used, making it a reliable downstream clustering tool in scRNA-seq analysis pipelines.

Clustering performance is evaluated both visually and quantitatively. For visualization, we use UMAP [19] plots to display the clustering structure in low-dimensional space. For quantitative evaluation, we use two metrics: Adjusted Rand Index (ARI) [24], which measures agreement between predicted and underlying true cluster labels, and compactness [3], which quantifies separation between true clusters. Higher ARI values reflect stronger agreement between predicted and true cluster labels, while higher compactness values suggest better separation between true clusters. Detailed definitions of these metrics are provided in Section 2.5.

**Figure 2:**
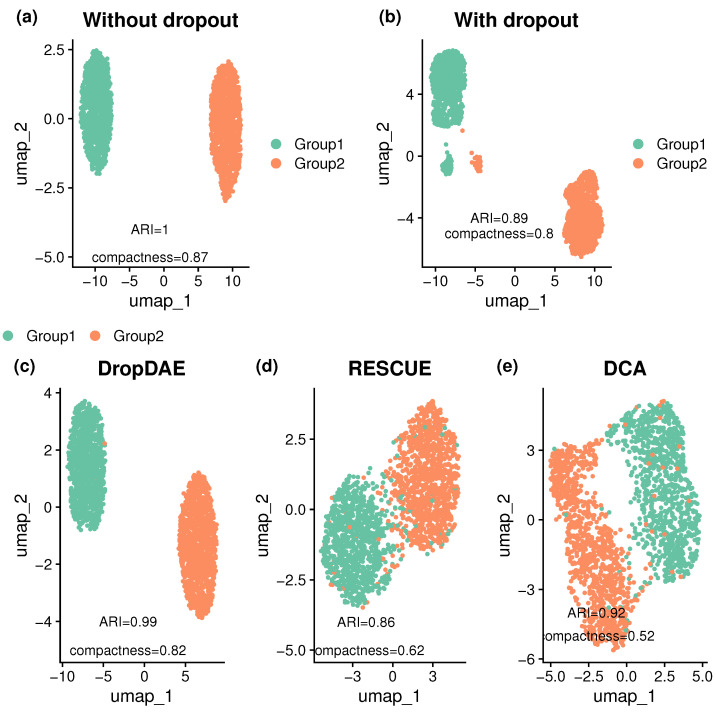
UMAP plots of simulated data before and after imputation. The first and second rows denote the results before and after denoising or imputation, respectively. Specifically, UMAP plots are shown using (**a**) uncorrupted data of ground truth without dropouts; (**b**) corrupted data with dropouts; (**c**) denoised data after applying DropDAE; (**d**) imputed data after applying RESCUE; (**e**) denoised data after applying DCA. ARI and compactness are calculated to assess clustering recovery. DropDAE achieves the highest ARI and compactness, demonstrating the strongest ability to recover the underlying biological clustering structure.

We first present results from the two-group simulation with moderate DE signals and moderate dropout events. Figure 2 illustrates UMAP plots of clustering outcomes before and after imputation. The ground truth data exhibit perfect separation (ARI = 1, compactness = 0.84), while the dropout-corrupted data show two small clusters due to dropouts, with a reduction in clustering performance (ARI = 0.89, compactness = 0.8).

Among the competing methods, DCA moderately recovers the underlying structure (ARI = 0.92, compactness = 0.52), while some cell types remain indistinct. RESCUE achieves even lower performance (ARI = 0.87, compactness = 0.64) than data with dropouts (ARI = 0.89, compactness = 0.8). Meanwhile, DropDAE achieves the best results, with an ARI of 0.99 and compactness of 0.82. This example demonstrates DropDAE’s ability to denoise the data and recover underlying biologically meaningful group structures.

To further evaluate DropDAE’s robustness under more complex biological scenarios, we extend our analysis to a six-group simulation setting with weak DE signals and moderate dropout level to make tasks more challenging. This setting reflects higher heterogeneity in cell populations and poses greater challenges for imputation and clustering.

**Figure 3:**
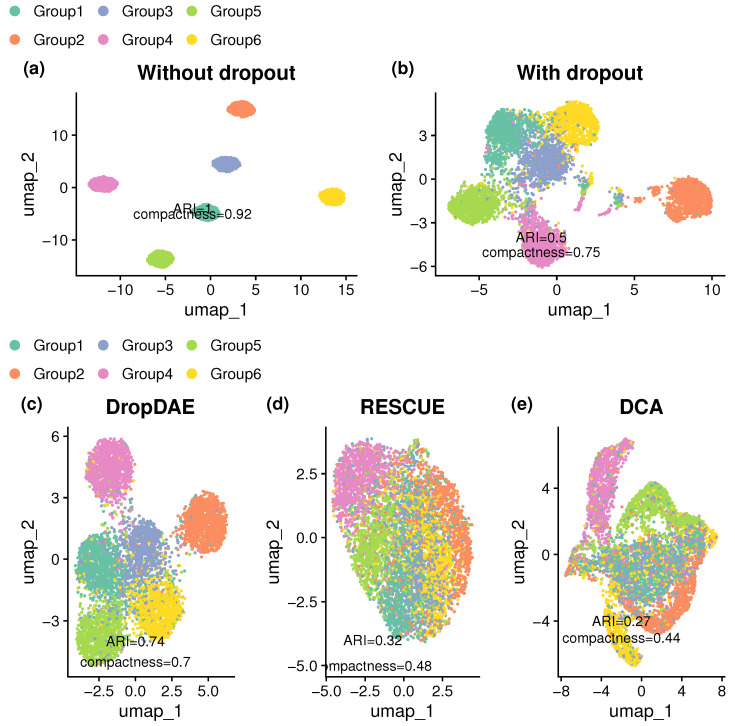
UMAP plots of simulated six-group data before and after imputation. The first and second rows denote the results before and after denoising or imputation, respectively. Specifically, UMAP plots are shown using (**a**) uncorrupted data of ground truth without dropouts; (**b**) corrupted data with dropouts; (**c**) denoised data after applying DropDAE; (**d**) imputed data after applying RESCUE; (**e**) denoised data after applying DCA. DropDAE with contrastive learning provides the most accurate recovery of the original group structure. ARI and compactness are shown for each method.

Figure 3 presents UMAP visualizations of the clustering results. As expected, the data with dropouts significantly distort the six-group structure (ARI = 0.5, compactness = 0.75), and DCA and RESCUE achieve even worse performance (ARI = 0.27 and 0.33, respectively), with many overlapping and contaminated cells. DropDAE achieves the highest ARI of 0.74 and compactness of 0.7 among competing methods, clearly recovering from dropout events and separating the six clusters. These findings further show the ability of DropDAE to restore complex clustering structures, especially in more challenging settings with weaker biological signals and greater biological variability.

### 3.2. Dropdae Exhibits Robustness Under Various Scenarios

To demonstrate the robustness of DropDAE in various simulation settings, we evaluate its performance under a total of nine conditions derived from all combinations of three levels of DE signals (weak, moderate, and strong) and three levels of dropout severity (weak, moderate, and strong). For each condition, we simulate two- and six-group datasets to assess performance under different degrees of complexity. Detailed parameter specifications are given in Section 2.3.

For each of the 9 simulation settings, we generate 100 independent datasets and apply DropDAE, RESCUE, and DCA. We assess the performance in clustering using ARI and compactness. The mean and standard deviation of each metric are computed over 100 independent datasets, and results are summarized by averaging these values across the 9 settings. Table 2 reports the summary statistics, demonstrating that DropDAE consistently outperforms competing methods in all evaluation metrics while maintaining low variance across nine settings. We also show the boxplots of ARI and compactness under different settings separately in Supplementary Figures S1–S4.

### 3.3. DropDAE Helps Cell Type Identification in the PBMC Dataset

To demonstrate the performance of DropDAE in real-world data, we download the Peripheral Blood Mononuclear Cells (PBMC) dataset, publicly available from 10X Genomics (Pleasanton, CA, USA) by https://cf.10xgenomics.com/samples/cell/pbmc3k/pbmc3k_filtered_gene_bc_matrices.tar.gz, accessed on 5 June 2025. It includes a variety of immune cell types and is suitable to evaluate DropDAE’s ability to denoise datasets with large heterogenity. The dataset contains 26,286 genes and 2700 cells, including naive and memory CD4+ T cells, CD8+ T cells, B cells, natural killer (NK) cells, CD14+ monocytes, FCGR3A+ monocytes, dendritic cells (DC), and platelets. To reduce the influence of rare cell populations, we exclude two minor clusters—DC and platelet—which together comprise only 46 cells. This decision is motivated by the observation that common clustering algorithm SNN is sensitive to small clusters and may misrepresent their structure in the presence of dominant populations. Additionally, we merge “Memory CD4 T cells” and “Naive CD4 T cells” into a single group, “CD4 T cells”. This step avoids over-fragmentation in clustering analysis, reduces redundancy, and improves interpretability by consolidating functionally similar subsets of CD4 T cells.

We preprocess the gene expression matrix using the SCTransform workflow implemented in the Seurat package. SCTransform performs variance-stabilizing transformation and normalizes for sequencing depth while modeling technical noise, resulting in a more reliable feature matrix for downstream tasks. After SCTransform, 12,572 highly variable features are retained. These features represent genes with stable and biologically informative signal across cells, serving as the effective input for dimensionality reduction, clustering, and denoising.

This dataset is highly sparse, with 93.5% of the original counts being zeros—a typical level of sparsity for scRNA-seq data [25,26]. Since we cannot determine whether the zero counts are real dropout events, the underlying ground truths without dropouts remain unknown. Hence, we introduce extra dropouts to the data and use them as input for all methods. The extra dropouts lead to an extra 4.5% of the zero counts, which makes the denoising even more challenging. After applying denoising or imputation methods, we can then evaluate how well the data with extra dropouts can be recovered to the original data without extra dropouts.

Similar to the simulation studies, we simulate 100 independent and identically distributed (i.i.d.) dropout patterns from the R Splatter package and add to the original data to serve as input for the denoising or imputation methods. These repeated simulations provide a robust framework to assess the accuracy and stability of DropDAE and other competing methods.

**Figure 4:**
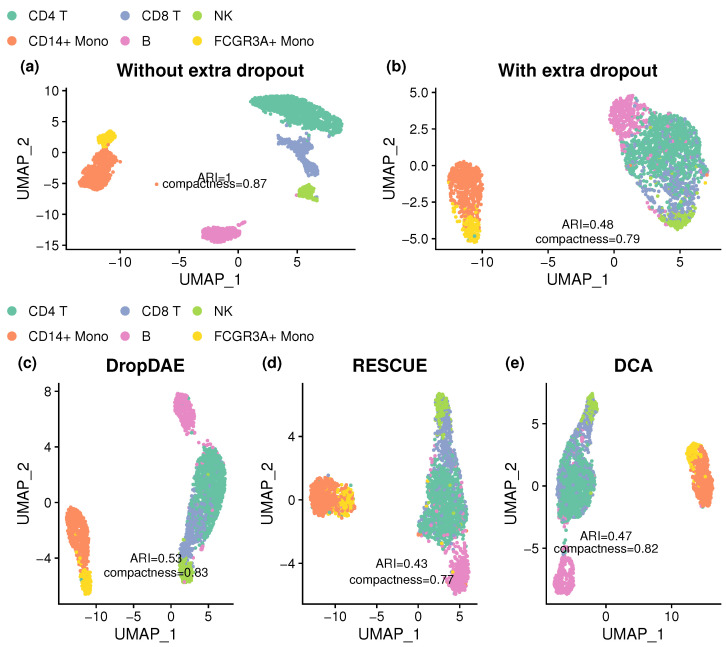
UMAP plots of the data with and without imputation or denoising to show the clustering patterns using the PBMC dataset. The first and second rows denote the results before and after denoising or imputation, respectively. Specifically, UMAP plots are shown using (**a**) uncorrupted real data without extra dropouts; (**b**) corrupted real data with extra dropouts; (**c**) denoised data after applying DropDAE; (**d**) imputed data after applying RESCUE; (**e**) denoised data after applying DCA. ARI and compactness are calculated based on the predicted clustering outcomes compared with the annotated clustering labels. Data without dropout show clear separation, while data with dropout significantly reduce the clustering pattern. The competing methods struggle with extra dropouts, while DropDAE restores the clustering pattern and outperforms other methods, with the highest ARI and compactness.

Figure 4 presents UMAP plots to demonstrate DropDAE’s effectiveness in recovering the clustering structure from the PBMC dataset after introducing extra dropouts. The original data without extra dropouts display a clear separation, with only slight indistinction between the CD14+Mono group and the FCGR3A+ Mono group, as reflected by an ARI of 1 and a compactness of 0.87. After introducing extra dropouts, the clustering pattern becomes obscured, resulting in a significant drop in clustering accuracy (ARI = 0.48) and reduced compactness (0.79). Competing methods including RESCUE and DCA struggle with the extra dropouts and show minor or no improvements from data with extra dropouts. In contrast, DropDAE more effectively restores the complicated clustering structure. In addition to detaching B cells from CD4 T, CD8 T, and NK cells as competing methods do, DropDAE also separates the CD14+Mono cluster and FCGR3A+ Mono cluster further, and it shows fewer overlapping cells among three clusters (CD4 T, CD8 T, NK). DropDAE achieves the highest ARI (0.57) and compactness (0.83), outperforming competing methods.

We further evaluate the robustness of DropDAE and competing methods using 100 repeats with independently simulated dropout patterns, focusing on numerical measures including ARI and compactness. Table 3 presents a summary table of the performance in clustering analysis across methods. Similarly to the example shown in Figure 4, manually introducing extra dropouts leads to a large reduction in ARI (from 1 to 0.475, on average). DropDAE consistently outperforms both DCA (*p*-value =0.0009074 for ARI, *p*-value =0.01567 for compactness) and RESCUE (*p*-value $=7.943\times 10^{-9}$ for ARI, *p*-value =0.0001684 for compactness) in both metrics with smaller variation in compactness. DCA and RESCUE achieve even lower means of ARI and compactness than data with dropouts, demonstrating poorer recovery of competing methods.

These results underscore DropDAE’s effectiveness in denoising real-world scRNA-seq data with complex, multi-group structures. The PBMC dataset presents a particularly challenging scenario due to its heterogeneous cell populations, high dimensionality of gene expression profiles, and relatively small number of cells. Despite these difficulties, DropDAE demonstrates superior clustering accuracy and robustness, making it a reliable tool for handling dropout events in real-world scRNA-seq data analyses.

## 4. Discussion

DropDAE addresses dropout events in scRNA-seq data by innovatively combining the architecture of DAE with contrastive learning. While DAE has been widely applied for handling noise and recovering underlying data structures [27], the integration of contrastive learning represents a significant advancement to further improve clustering. Traditionally, contrastive learning has been used in supervised contexts [17], where true labels guide the separation of instances. In DropDAE, we extend contrastive learning to an unsupervised approach by generating pseudo-labels through clustering the low-dimensional representations, enabling enhanced group separation without relying on ground truth information. The DAE component reconstructs corrupted gene expression profiles, while the contrastive learning component explicitly reinforces meaningful clustering structures in the latent space through triplet loss, making DropDAE a useful tool for denoising scRNA-seq data and improving downstream clustering analyses.

One of the key advantages of DropDAE lies in its flexibility and adaptability. Leveraging a relatively straightforward neural network architecture, with innovative loss functions (MSE and triplet loss), DropDAE can be easily customized by adjusting components such as network depth, width, activation functions, or loss function weighting to suit specific dataset characteristics and analytical goals. Most importantly, DropDAE exhibits the high accuracy and robustness in clustering performance, as demonstrated by comparative analyses across comprehensive simulation settings and a real-world dataset.

In addition, DropDAE offers a computational advantage over many existing methods. Most existing methods for addressing dropouts, such as RESCUE, are neighbor-based and rely on graph construction or sampling procedures to estimate local gene expression structure. These steps are computationally expensive and scale poorly with large datasets. In contrast, DropDAE avoids neighborhood sampling, making it faster and more scalable for large-scale single-cell applications.

Furthermore, compared to DCA—a competing deep learning method that also employs an autoencoder—DropDAE adopts a simpler yet more flexible design. DCA explicitly models gene expression using a zero-inflated negative binomial (ZINB) distribution, requiring estimation of the entire probability distribution. In contrast, DropDAE avoids the need to model such complex distributions. It reconstructs gene expression directly from corrupted inputs using a denoising autoencoder, thereby reducing model complexity and improving generalizability. In addition, DropDAE uniquely integrates contrastive learning to enhance the separation of biologically distinct cell populations, further improving clustering performance. Together, these features reduce model complexity, avoid strong distributional assumptions, and offer greater flexibility across diverse single-cell datasets.

However, DropDAE also shares some common challenges observed in deep learning-based methods. The black-box nature of neural networks can limit interpretability [28], making it harder to trace specific biological insights directly from the model. Furthermore, tuning hyperparameters, such as the influence of triplet loss λ and margin α, is crucial but challenging. These parameters are important for balancing accurate reconstruction of gene expression profiles and effective separation of cell groups, but they can vary in different datasets. Proper tuning often requires extensive validation, reflecting a broader challenge commonly faced by deep learning models in biological data analysis.

## 5. Conclusions

In this work, we propose DropDAE, a denoising autoencoder framework enhanced with contrastive learning, specifically designed to address dropout events in scRNA-seq data. By combining MSE loss with triplet loss, DropDAE simultaneously reconstructs corrupted gene expression profiles and improves clustering separation, producing robust and biologically meaningful representations.

We comprehensively evaluated DropDAE through extensive simulation studies. In scenarios involving either two or six underlying cell clusters, and across nine distinct combinations of differential expression signal strength and dropout level, DropDAE consistently outperformed competing methods in clustering analysis, with evidence in both dimension reduction plots and numerical measures including ARI and compactness.

Beyond simulations, we also applied DropDAE to a real-world scRNA-seq dataset, which presents practical challenges such as unknown underlying structures and highly heterogeneous dropout patterns. Even under these more complex conditions, DropDAE still achieved better clustering compared to RESCUE and DCA.

Looking ahead, here are promising directions for extending DropDAE. Given the flexibility and generalizability of denoising autoencoders, DropDAE could be adapted to other types of omics data or high-dimensional datasets that require denoising. Moreover, the integration of contrastive learning with DAE architectures appears promising and useful to improve clustering quality in complex datasets.

Overall, DropDAE offers a principled and flexible framework for denoising scRNA-seq data and improving downstream analyses, with greater potential for applications in a wide range of high-dimensional complex datasets.

## Figures and Tables

**Table 1:** Simulation parameters for generating scRNA-seq data using the R package *Splatter*.

Parameter	Interpretation	Value
seed	Random seed	Index of iteration
ngroups	Number of groups	2
batchCells	Cells per batch	5000
nGenes	Number of genes	500
de.prob	DE probability	0.1
de.facLoc	DE factor location	0.1 (weak signal)
		0.2 (moderate signal)
		0.3 (strong signal)
de.facScale	DE factor scale	0.4
de.downProb	Down-regulation probability	0.5
dropout.type	Dropout type	“experiment”
dropout.mid	Dropout mid point	2 (weak dropout)
		4 (moderate dropout)
		5 (strong dropout)
dropout.shape	Dropout shape	−1

**Table 2:** Summary of the averaged ARI and compactness for clustering analysis under nine simulation settings. For each setting, 100 independent datasets are generated, and ARI and compactness are computed. Values are presented as the average of the means, and values in the parenthesis are the average of the standard deviations from nine settings.

Method	Two Groups		Six Groups	
	ARI	Compactness	ARI	Compactness
Without dropout	1 (0)	0.848 (0.015)	1 (0)	0.921 (0.004)
With dropout	0.837 (0.197)	0.551 (0.278)	0.705 (0.102)	0.435 (0.304)
DropDAE	0.924 (0.054)	0.729 (0.065)	0.81 (0.062)	0.783 (0.046)
DCA	0.542 (0.264)	0.45 (0.202)	0.596 (0.102)	0.484 (0.256)
RESCUE	0.709 (0.165)	0.513 (0.21)	0.483 (0.066)	0.301 (0.267)

**Table 3:** Summary of the ARI and compactness for clustering analysis using the PBMC dataset. For each setting, 100 corrupted datasets based on the original dataset with the same level of extra dropouts are generated, and the numerical measures are computed. Values are presented as the means, and values in the parenthesis are the standard deviations.

Method	ARI	Compactness
Without dropout	1 (0)	0.872 (0)
With dropout	0.475 (0.034)	0.806 (0.023)
DropDAE	0.558 (0.042)	0.825 (0.017)
RESCUE	0.414 (0.023)	0.778 (0.035)
DCA	0.486 (0.034)	0.803 (0.014)

## Data Availability

DropDAE is implemented in R and is available at https://github.com/wanlinjuan/DropDAE, accessed on 5 June 2025.

## Supplementary Materials

The following supporting information can be downloaded at: https://www.mdpi.com/article/10.3390/bioengineering12080829/s1, Figure S1: Boxplots of ARI under two-group setting using data without dropouts, data with dropouts, and imputed data using DropDAE or competing methods. Figure S2: Boxplots of compactness under two-group setting using data without dropouts, data with dropouts, and imputed data using DropDAE or competing methods. Figure S3: Boxplots of ARI under six-group setting using data without dropouts, data with dropouts, and imputed data using DropDAE or competing methods. Figure S4: Boxplots of compactness under six-group setting using data without dropouts, data with dropouts, and imputed data using DropDAE or competing methods.
