# Supplementary material for: DropDAE: Denosing Autoencoder with Contrastive Learning for Addressing Dropout Events in scRNA-seq Data
Source: Bioengineering (Basel). 2025 Jul 31;12(8):829. doi: 10.3390/bioengineering12080829 (PMC12383624; doi:10.3390/bioengineering12080829)
Supplement: Supplementary file 1 [file bioengineering-12-00829-s001.zip › bioengineering-3712897-supplementary.pdf]

DropDAE: Denosing Autoencoder with contrastive learning for Addressing Dropout Events in scRNA-seq Data

July 25, 2025

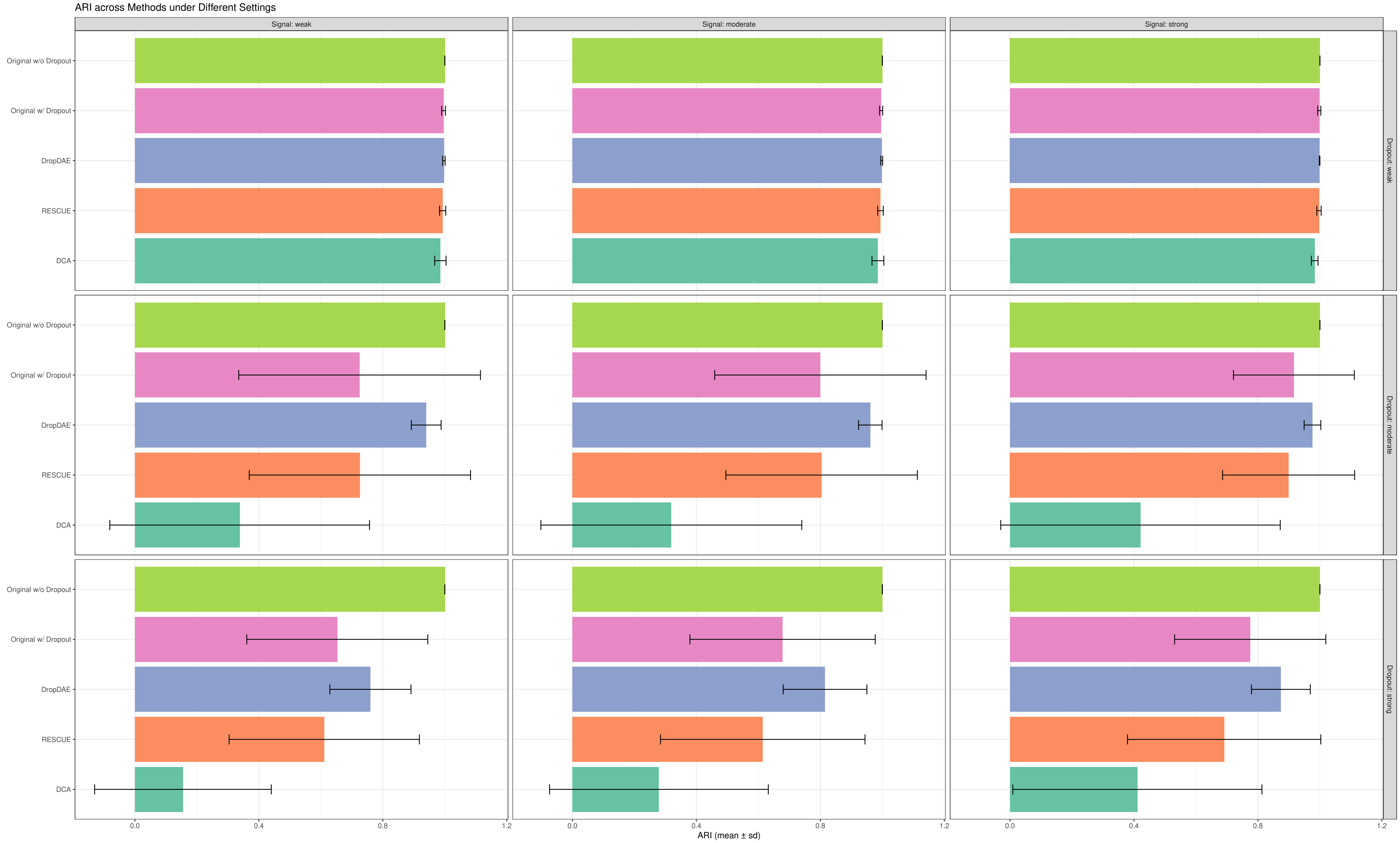

Supplementary Figure S1: Boxplots of ARI under two-groups setting using data without dropouts, data with dropouts and reconstructed data using DropDAE or competing methods.

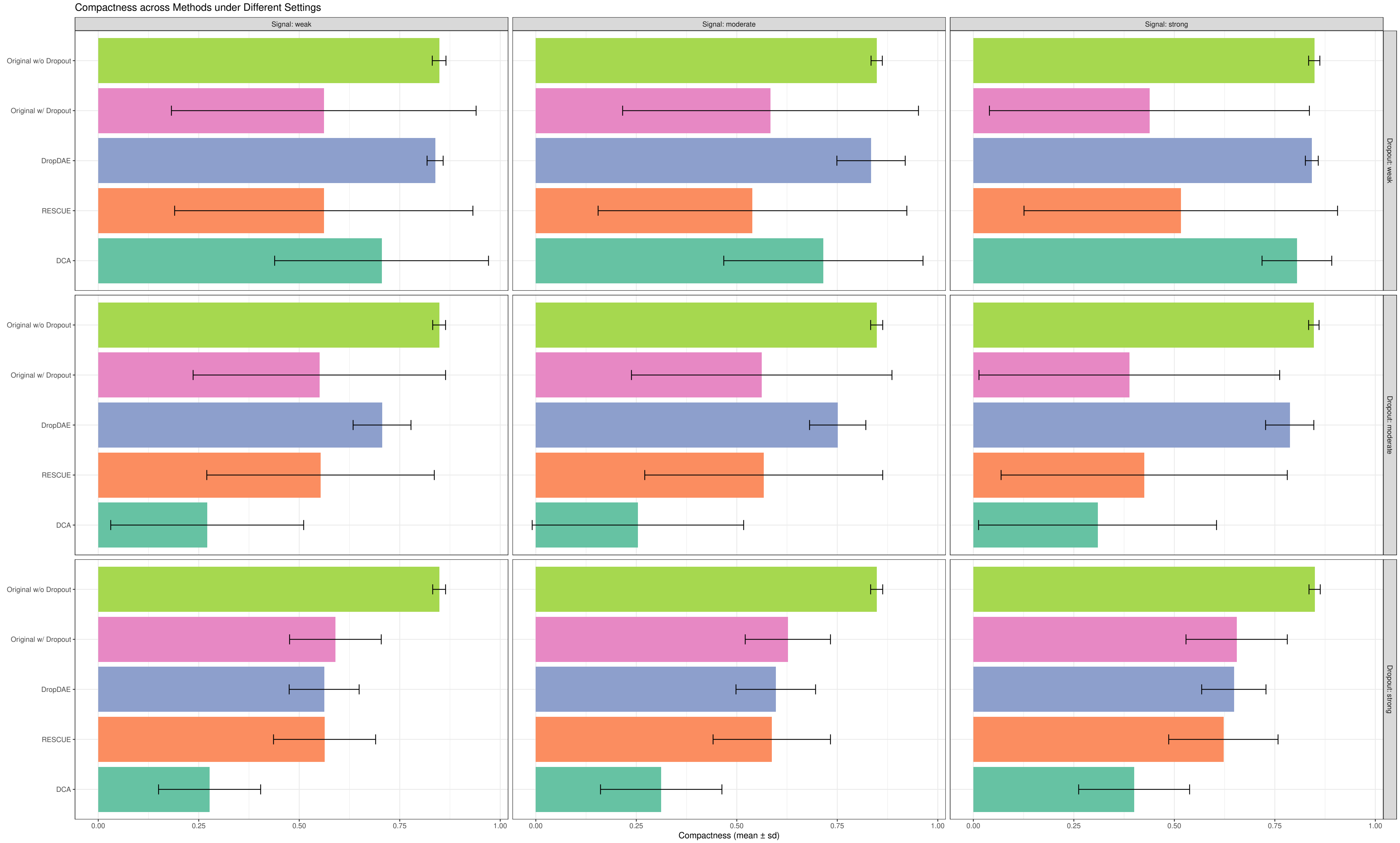

Supplementary Figure S2: Boxplots of compactness under two-groups setting using data without dropouts, data with dropouts and reconstructed data using DropDAE or competing methods.

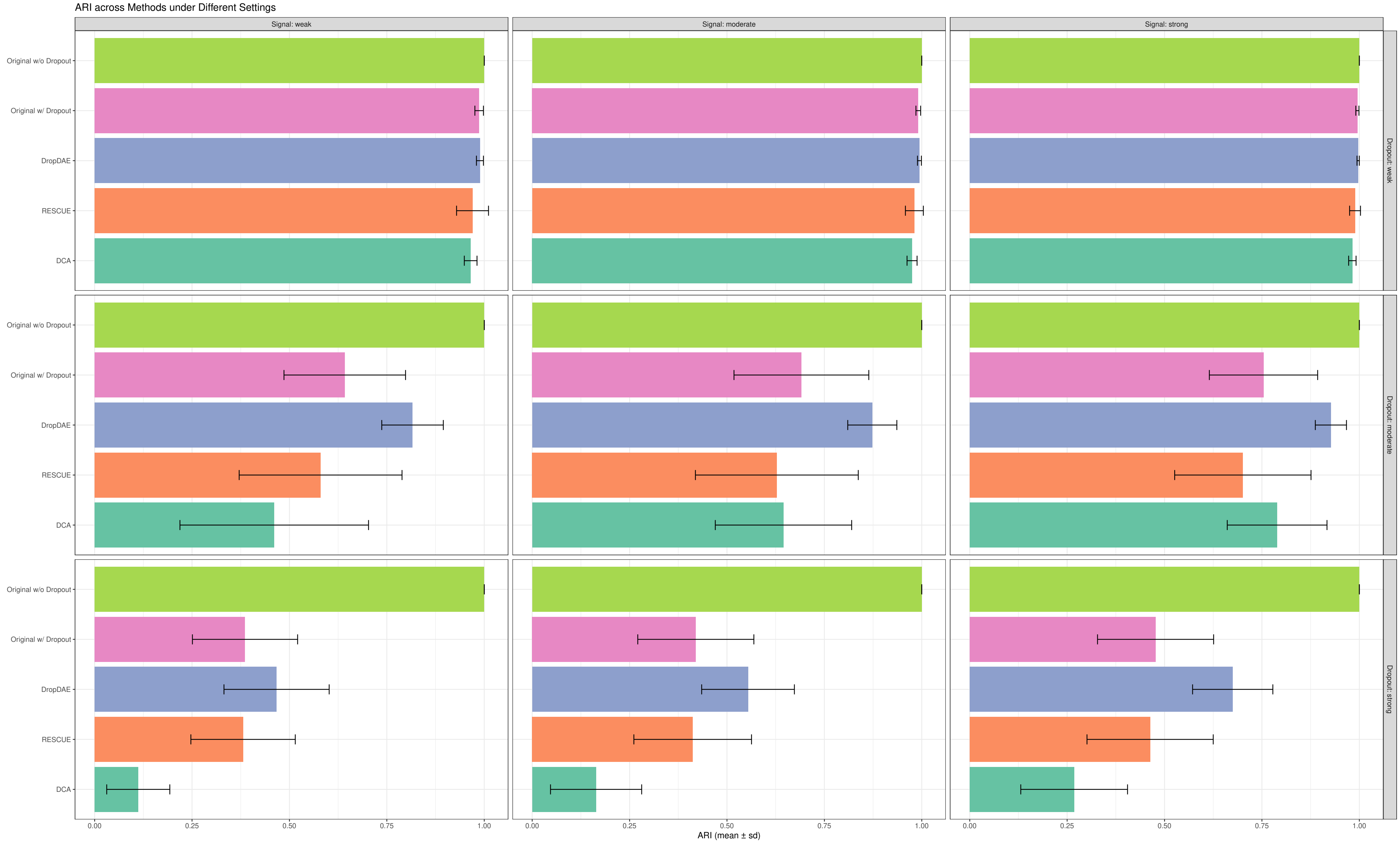

Supplementary Figure S3: Boxplots of ARI under six-groups setting using data without dropouts, data with dropouts and reconstructed data using DropDAE or competing methods.

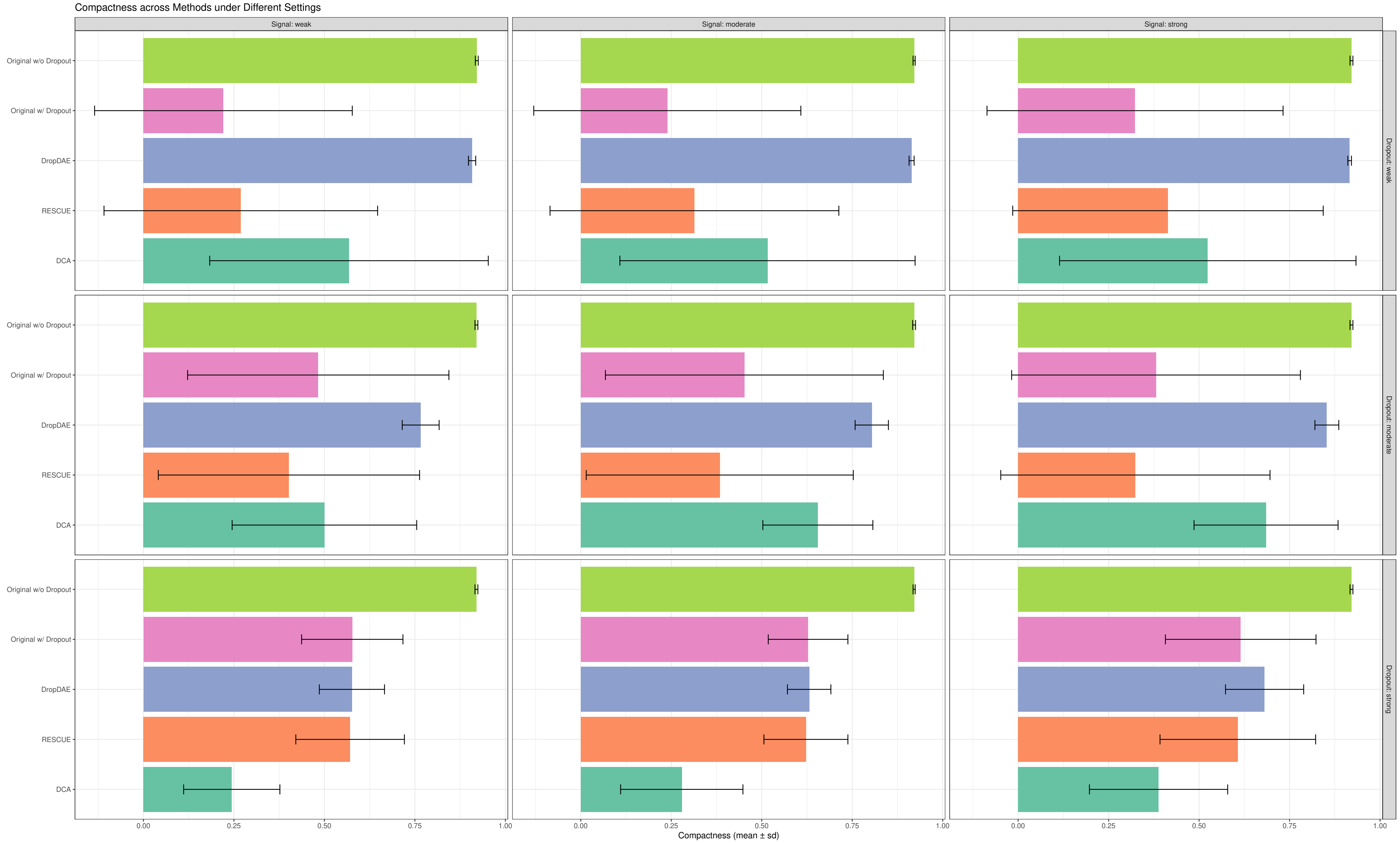

Supplementary Figure S4: Boxplots of compactness under six-groups setting using data without dropouts, data with dropouts and reconstructed data using DropDAE or competing methods.
